# Acute Valproate Exposure Induces Mitochondrial Biogenesis and Autophagy with FOXO3a Modulation in SH-SY5Y Cells

**DOI:** 10.3390/cells10102522

**Published:** 2021-09-23

**Authors:** Eun-Hye Jang, Jung-Ho Lee, Soon-Ae Kim

**Affiliations:** Department of Pharmacology, School of Medicine, Eulji University, Daejeon 34824, Korea; dmter12@gmail.com (E.-H.J.); jungholee@eulji.ac.kr (J.-H.L.)

**Keywords:** autophagy, FOXO3a, mitochondria, valproic acid

## Abstract

Valproic acid (VPA) is an antiepileptic drug found to induce mitochondrial dysfunction and autophagy in cancer cell lines. We treated the SH-SY5Y cell line with various concentrations of VPA (1, 5, and 10 mM). The treatment decreased cell viability, ATP production, and mitochondrial membrane potential and increased reactive oxygen species production. In addition, the mitochondrial DNA copy number increased after VPA treatment in a dose-dependent manner. Western blotting showed that the levels of mitochondrial biogenesis-related proteins (PGC-1α, TFAM, and COX4) increased, though estrogen-related receptor expression decreased after VPA treatment. Further, VPA treatment increased the total and acetylated FOXO3a protein levels. Although SIRT1 expression was decreased, SIRT3 expression was increased, which regulated FOXO3 acetylation in the mitochondria. Furthermore, VPA treatment induced autophagy via increased LC3-II levels and decreased p62 expression and mTOR phosphorylation. We suggest that VPA treatment induces mitochondrial biogenesis and autophagy via changes in FOXO3a expression and posttranslational modification in the SH-SY5Y cell line.

## 1. Introduction

Valproic acid (VPA) is used as an anticonvulsant and mood stabilizer. It is a branched medium-chain fatty acid known to inhibit mitochondrial β-oxidation and oxidative phosphorylation (OXPHOS), which may induce mitochondrial dysfunction [1,2]. VPA is also a histone deacetylase inhibitor that may play a role in epigenetic modification and protein posttranslational modification. Considering the mitochondrial dysfunction and VPA-induced epigenetic modification, cancer treatment with VPA has recently garnered attention [3]. VPA has shown potent antitumor effects in various in vitro and in vivo systems and encouraging results in early clinical trials, either alone or in combination with demethylating and/or cytotoxic agents [4]. VPA treatment has also been shown to induce autophagy in cancer cell lines [5,6]. Moreover, increasing evidence has linked forkhead box class O (FOXOs) to autophagy in human health and diseases [7]. Mammals have four FOXO proteins, namely FOXO1, 3, 4, and 6, which show high sequence similarity. These factors are ubiquitously expressed, and the expression level of each of these proteins is tissue-specific, reflecting potential differences in their cellular activity [8].

In humans, FOXO3a activity has been linked to increased lifespan through modulation of responses to various stresses, including oxidative stress. Notably, FOXO3a has been suggested as a core regulator of cellular homeostasis, stress response, and longevity [9]. Furthermore, functional alterations of FOXO3a have been correlated with poor prognosis in several types of cancer [10,11]. The main processes regulated by FOXO3a include autophagy [12] via reactive oxygen species (ROS) detoxification [13]. Recent studies have shown that FOXO3a is imported into the mitochondria in tumor cells and tissues subjected to metabolic stress and cancer therapeutics, where it promotes the expression of mitochondrial genes to support mitochondrial metabolism and cell survival [14].

Although several studies have evaluated the effects of VPA on neuroblastoma [15,16,17], there have been no studies on how VPA treatment affects FOXO3a and its mechanisms. Therefore, in this study, we tried to determine the effect of different concentrations of VPA on FOXO3a in the neuroblastoma cell line SH-SY5Y and on mitochondrial biogenesis and autophagy.

## 2. Materials and Methods

### 2.1. Cell Culture

The human neuroblastoma SH-SY5Y cell line was obtained from the Korean Cell Line Bank (Seoul, Korea). The cells were cultured in Eagle’s minimum essential medium (EMEM, Lonza, Basel, Switzerland) supplemented with 50% F-12 (Lonza, Basel, Switzerland), 10% fetal bovine serum (Alphabioregen, Boston, MA, USA), 1% penicillin and streptomycin (Gibco, Waltham, MA, USA), 1× non-essential amino acid (NEAA, Lonza, Basel, Switzerland), and 1 mM sodium pyruvate (Sigma-Aldrich, St. Louis, MO, USA). 

### 2.2. Cytotoxicity Test 

SH-SY5Y cells were plated in a 96-well cell culture plate at 1 × 10^5^ cells/100 μL and incubated in a 5% CO_2_ incubator at 37 °C for 24 h. After adhering sufficiently to the well, the cells were treated with VPA (0, 1, 5, and 10 mM) for 24 h. The VPA was purchased from Sigma (Sigma-Aldrich, St. Louis, MO, USA). The cytotoxicity of VPA was determined by adding 10 μL of cell counting kit-8 reagent (CCK-8; Dojindo, Kumamoto, Japan) to each well and incubated for 2 h in the dark. The absorbance of each well was then measured at 450 nm using a microplate photometer (Thermo Scientific, Waltham, MA, USA).

### 2.3. LDH Assay

The cytotoxicity was measured using the LDH cytotoxicity detection kit (Takara, Kusatsu, Japan). SH-SY5Y cells were plated in a 96-well cell culture plate at 1 × 10^5^ cells/100 μL and incubated in a 5% CO_2_ incubator at 37 ℃ for 24 h. After adding 100 μL of fresh medium, we treated VPA (0, 1, 5, and 100 mM) or VPA plus 100 nM bafilomycin A1 for 24 h. Cells were treated with 1% Tripton X-100 for 10 min and the supernatant was used as a positive control. After centrifuging the cells at 250× *g* for 10 min, we transferred 50 μL of the supernatant to a new plate. The solution (100 μL) contained in the kit and fresh medium (50 μL) was added to the supernatant and reacted for 30 min after blocking the light. The absorbance of each well was then measured at 490 nm using a microplate photometer (Thermo Scientific, Waltham, MA, USA). The cytotoxicity was calculated according to the kit’s instructions.

### 2.4. ATP Lite Assay

ATP concentrations were measured using an ATP Lite Kit (PerkinElmer, Waltham, MA, USA). The luciferase and D-luciferin included in the kit emit light by reacting with ATP, which is proportional to the ATP concentration. SH-SY5Y cells were seeded at 5 × 10^4^ cells/well in 96-well plates and incubated for 24 h. The cells were then treated with various concentrations (0, 1, and 5 mM) of VPA for 4 h. Next, 50 μL of mammalian cell lysis solution was added to each well and the plate was shaken at 150 rpm for 5 min. The substrate solution (50 μL) was added to each well; the mixture was protected from light for 10 min, and luminescence was measured using a multimode detector (Beckman Coulter, Brea, CA, USA).

### 2.5. Mitochondrial Membrane Potential Assay

SH-SY5Y cells were plated at 5 × 10^4^ cells/well in a 96-well black plate and incubated for 24 h. The cells were then treated with various concentrations of VPA for 4 h. Mitochondrial membrane potential was measured using the JC-10 Mitochondrial Membrane Potential Assay Kit (Abcam, Cambridge, UK). When the membrane potential was low, JC-10 remained a monomer emitting green light. If the membrane potential was high, the aggregated JC-10 monomers emitted red light. Fifty microliters of JC-10 reagent were added to each well, and the plate was incubated for 1 h at 37 °C and 5% CO_2_ in a dark chamber. Fluorescence intensity was detected using a multimode detector (Beckman Coulter, Brea, CA, USA) at Ex/Em = 490/525 nm and Ex/Em = 540/590 nm. Changes in mitochondrial membrane potential were determined as the ratio between the aggregate (Em 525 nm) and monomeric forms (Em 590 nm) of JC-10.

### 2.6. Oxidative Stress Analysis

To determine the effect of VPA treatment on oxidative stress, the assay was performed using the Muse^®^ Oxidative Stress Kit (Millipore, Burlington, MA, USA). SH-SY5Y cells were plated at 1 × 10^6^ cells/mL in a 6-well plate and incubated in a 5% CO_2_ incubator at 37 °C for 24 h. The cells were treated with VPA (0, 1, 5, and 10 mM) for 24 h and then harvested by trypsinization, centrifuged, washed with phosphate-buffered saline, pelleted, and resuspended in 1 × assay buffer at 1 × 10^6^ to 1 × 10^7^ cells/mL. The cell suspension (10 μL) was mixed with 190 μL of working solution and incubated at 37 °C for 30 min. The working solution was prepared by diluting the kit’s reagent in 1× assay buffer, as described by the manufacturer. The mixtures were vortexed for 3–5 s and analyzed using a Muse^TM^ cell analyzer (Millipore, Burlington, MA, USA).

### 2.7. Mitochondrial (mt) DNA Copy Number Assay

For DNA isolation, 1 × 10^6^ SH-SY5Y cells were seeded onto a 100 mm dish for 24 h. Total cellular DNA was extracted using the DNeasy Blood and Tissue Kit (Qiagen, Hilden, Germany). qRT-PCR was performed using the iQTM SYBR^®^ Green Supermix (Bio-Rad, Hercules, CA, USA) in a CFX96TM Real-Time system (Bio-Rad, Hercules, CA, USA). The target genes were amplified under the following conditions: 95 °C for 10 min, 40 cycles of 95 °C for 15 s, and 60 °C for 1 min. The relative mtDNA copy number was determined by the 2^−^^ΔΔCt^ method [18], using the equation: Relative mtDNA copy number = 2^−ΔCt^, where ΔCt= Ct^mitochondrial^ − Ct^nuclear^. Pyruvate kinase (PK) genes were used as nuclear DNA (nDNA). The cytochrome b (CYTB) and NADH dehydrogenase subunit 1 (ND1) and subunit 4 (ND4) genes were used as representatives of mtDNA in the samples. The sequences of primers used for amplification of the target genes are included in Supplementary Table (Table S1).

### 2.8. Western Blotting 

For quantification of protein expression, SH-SY5Y cells were seeded at a density of 1 × 10^6^ cells in 100 mm dish. After 24 h, the cells were treated with 0, 1, and 5 mM VPA for 24 h. Cell proteins were extracted using RIPA buffer (ATTO, Tokyo, Japan) with proteinase and phosphatase inhibitors (ATTO, Tokyo, Japan). Nuclear proteins of cells were isolated using NEPER kit (Thermo scientific, Baton Rouge, LA, USA). Protein concentration was measured using a BCA assay (Thermo Scientific, Waltham, MA, USA). Equal amounts (20 μg) of protein samples were separated using 10% SDS-PAGE and transferred to nitrocellulose membranes (Pall, Port Washington, NY, USA). The membranes were blocked with 5% non-fat milk in TBST buffer for 1 h at room temperature and incubated with primary antibodies at 4 °C overnight. The following primary antibodies were used: anti-p-mTOR, anti-mTOR, anti-LC3A/B, anti-COX4, anti-ERRα, anti-SIRT1, anti-SIRT3, anti-FOXO3a, anti-TFAM, anti-β-actin, anti-Lamin-B1 (Cell Signaling Technology, Danvers, MA, USA), anti-PGC-1α (Millipore, Burlington, MA, USA), and anti-p62 (Abcam, Cambridge, UK). The membranes were rinsed with TBST buffer and incubated with HRP-labeled rabbit or mouse secondary antibodies (Invitrogen, Carlsbad, CA, USA) for 1 h. After washing with TBST buffer, the membranes were incubated with West Femto Maximum Sensitivity Substrate (Thermo Scientific, Waltham, MA, USA). The amount of protein expression was detected by exposure of the membrane to an X-ray film (Agfa, Mortsel, Belgium) and analyzed using ImageJ software version 1.53a (NIH, Bethesda, MD, USA).

### 2.9. Immunocytochemistry

A total of 1.5 × 10^6^ SH-SY5Y cells were seeded in the camber slide and incubated in a 5% CO_2_ incubator at 37 °C for 24 h. We treated VPA (0, 1, 5, and 10 mM), 100 nM bafilomycin A1, or 5 mM VPA plus 100 nM bafilomycin A1 to the SH-SY5Y cells for 24 h. The cells were fixed with PBS containing 2% formaldehyde and 2.5% glutaraldehyde for 10 min, washed three times with 1× PBS. Blocking was performed using a blocking solution (PBS with 0.1% Tween 20 and 1% bovine serum albumin) at room temperature for 1 h. The cells treated with VPA (0, 1, 5 and 10 mM) were incubated with anti-FOXO3a primary antibody (Cell Signaling Technology, Danvers, MA, USA) for 1 h at room temperature. The cells treated with VPA (0, 5 mM), 100 nM bafilomycin A1, or 5 mM VPA plus 100 nM bafilomycin A1 were incubated with anti-LC3A/B primary antibody (Cell Signaling Technology, Danvers, MA, USA) for 1 h at room temperature. After washing three times with 1 × PBS, the cells were incubated with Alexa Fluor 594-conjugated IgG secondary antibody (Abcam, Cambridge, UK) in the dark for 1 h at room temperature. After washing three times with 1 × PBS, the cells were mounted using Fluoroshield mounting solution (Abcam, Cambridge, UK). A fluorescence microscope (Korea Lab Tech, Seongnam, Gyeonggi, Korea) was used for image confirmation.

### 2.10. Immunoprecipitation

We added VPA (0, 5 mM) into SH-SY5Y cells, which were seeded in a 100 mm dish at a density of 1 × 10^7^ cells for 24 h. The cells were harvested by trypsinization, centrifuged, washed with DPBS, and pelleted. The total protein of the SH-SY5Y cell pellet was extracted with lysis buffer included in the PierceTM Direct IP Kit (Thermo Scientific, Waltham, MA, USA) and nucleic protein was isolated using the NEPER Kit (Thermo Scientific, Waltham, MA, USA). Total protein from the VPA model hippocampus region was isolated using RIPA buffer (ATTO, Tokyo, Japan). Following the manufacturer’s instructions, the impurities in the protein extract were cleared using a column contained in the kit. Then, a new column, with the resin contained in the kit, and anti-FOXO3a antibody (Abcam, Cambridge, UK) were used. In the final step, each sample was obtained by responding to the cleared extracts with antibody added to the column. The sample was mixed with 2× sample loading buffer (LPS solution, Daejeon, Korea) and boiled for 5 min at 100 °C. Western blotting was performed according to a previously described method. The primary antibody used was rabbit anti-acetyl lysine (Abcam, Cambridge, UK), while Foxo3a was used as the loading control.

### 2.11. Autophagy Detection

SH-SY5Y cells were seeded in the camber slide at 1.5 × 10^6^ cells/500 μL or in a 96-well cell culture plate at 1 × 10^5^ cells/100 μL. The cells were incubated in a 5% CO_2_ incubator at 37 ℃ for 24 h. The cells in the chamber slide were treated with VPA (0, 5 mM), 100 nM bafilomycin A1, or 5 mM VPA plus 100 nM bafilomycin A1. The cells in the 96-well cell culture plate were treated with VPA (0, 1, 5, and 10 mM) alone or together with 100 nM bafilomycin A1. After 24 h, autophagy was measured using an autophagy detection kit (Abcam, Cambridge, UK). After removing the medium from cells, the detection reagent included in the kit was treated and reacted in the dark for 30 min. The fluorescence intensity (Ex/Em = 485/585) of the 96-well cell culture plate was measured using a multimode detector (Beckman Coulter, Brea, CA, USA).

### 2.12. Statistical Analysis

All data are expressed as the mean ± standard deviation. Statistical analyses were performed by one-way ANOVA followed by Tukey’s post hoc test or unpaired *t*-test using SPSS v20 (IBM, Armonk, NY, USA). Significant *p*-value data are denoted in the graphs as follows: * *p* < 0.05, ** *p* < 0.01, and *** *p* < 0.001.

## 3. Results

### 3.1. VPA Induced Cytotoxicity with Changes to the Mitochondria Membrane Potential and ATP Production in the SH-SY5Y Cell Line 

**Figure 1:**
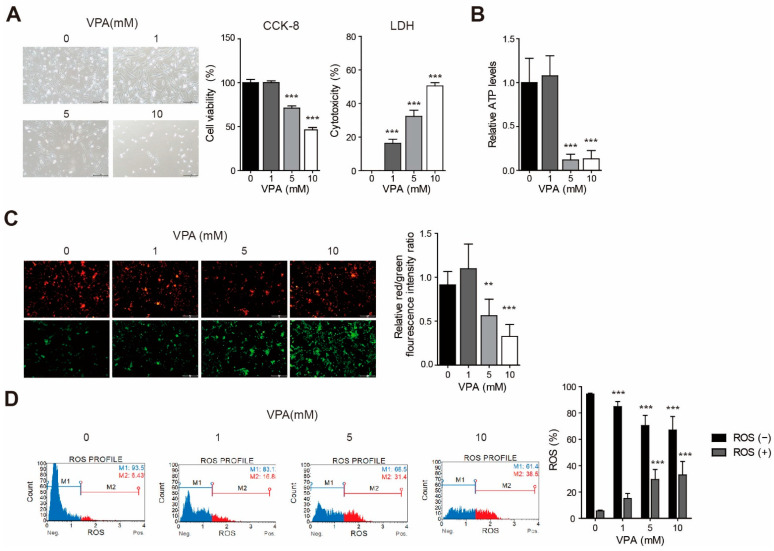
Valproic acid (VPA) induced cytotoxicity with a change to mitochondria membrane potential and ATP production in the SH-SY5Y cell line. SH-SY5Y cells were treated with various concentrations (0, 1, 5, or 10 mM) of VPA. (**A**) Cell viability and cytotoxicity were measured in cells treated with VPA for 24 h using the CCK-8 assay and the LDH assay (*n* = 6). Scale bar = 100 μm. (**B**) Cells treated with VPA for 4 h were lysed and the ATP level was measured (*n* = 6). (**C**) The JC-10 kit was used to observe changes in mitochondrial membrane potential (MMP) in cells treated with VPA for 4 h. At low MMP, JC-10 predominantly exists as a monomer that yields green fluorescence. At high MMP, JC-10 aggregates yield red fluorescence (*n* = 9). Scale bar = 100 μm. (**D**) ROS-producing cells treated with VPA for 24 h (*n* = 6). Values represent the mean ± SD. ** *p* < 0.01, and *** *p* < 0.001 with the control (0 mM group).

We treated the SH-SY5Y cell line with various concentrations of VPA. Microscopic observations revealed that VPA treatment induced a cytotoxic phenotype (Figure 1A). We observed a decrease in the number of cells in the high-power field in the SH-SY5Y cell line and the shortening of neurite length in 5 mM VPA treatment for 24 h. Cell viability and toxicity were measured using the CCK-8 assay and the LDH assay. At concentrations of 5 mM and 10 mM, cell viability is significantly reduced (Figure 1A; *p* < 0.001) in the CCK-8 assay result. The LDH assay result showed that the cytotoxicity significantly increased when we treated VPA at a concentration of 1 mM or higher (Figure 1A; *p* < 0.001 at 1, 5, 10 mM). Treatment with 5 mM and 10 mM VPA for 4 h significantly inhibit ATP production (Figure 1B; *p* < 0.001 at 5 mM and 10 mM). After 4 h of VPA treatment at concentrations above 5 mM, mitochondrial membrane potential decreases significantly (Figure 1C; *p* < 0.01 at 5 mM; *p* < 0.001 at 10 mM). When the flow cytometric assay using the Muse cell analyzer was used, the proportion of cells producing ROS increased with the VPA treatment, and the proportion of cells not producing ROS decreased, which appeared to be dose-dependent (Figure 1D).

### 3.2. Change of mtDNA Copy Numbers and Protein Levels for Mitochondria Biogenesis-Related Genes with VPA Acute Exposure in the SH-SY5Y Cell Line 

**Figure 2:**
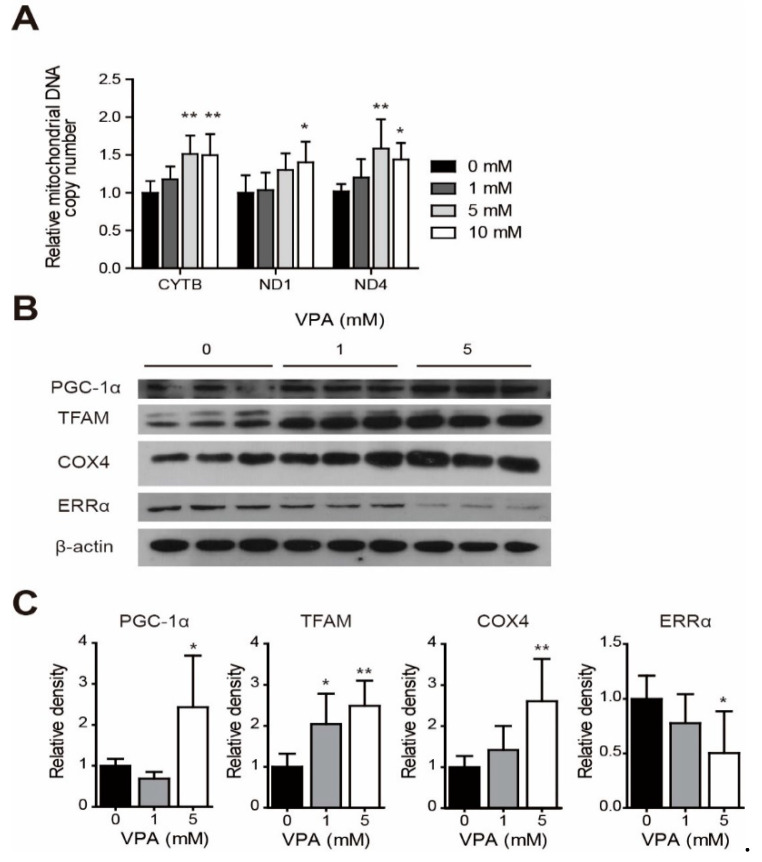
Change to the mtDNA copy number and protein levels for mitochondria biogenesis-related genes after acute VPA exposure in SH-SY5Y cell line. (**A**) Total genomic DNA was isolated from SH-SY5Y cells treated with different concentrations (0, 1, 5, and 10 mM) of VPA for 72 h, and the mitochondrial DNA (mtDNA) copy number was measured using qRT-PCR. The mtDNA copy number was analyzed by comparing the nDNA (PK) and mtDNA (CYTB, ND1, ND4). (**B**) Proteins were isolated from SH-SY5Y cells treated with various concentrations (0, 1, and 5 mM) of VPA for 24 h, and the expression of the target protein was observed by Western blot. (**C**) The level of protein expression was calculated by analyzing the band of (**B**) with the image J program and normalized by the β-actin protein expression level. Values represent the mean ± SD (*n* = 6). * *p* < 0.05 and ** *p* < 0.01 with the control group.

To assess the effect of VPA on mtDNA copy number, we compared the relative mtDNA copy numbers in the SH-SY5Y cells after 72 h of treatment. The ratios of ND1 gene in mtDNA to nDNA (PK) increases after treatment with 10 mM VPA (Figure 2A, *p* < 0.05). The ratio of mtDNA to nDNA (PK) of the CYTB gene and ND4 gene also increases significantly at concentrations higher than 5 mM (Figure 2A; CYTB, *p* < 0.01 at 5 mM and 10 mM; ND4, *p* < 0.01 at 5 mM, *p* < 0.05 at 10 mM). VPA treatment may alter the protein expression of mitochondrial biogenesis-related genes. To investigate whether acute VPA exposure affects mitochondria-related gene expression in the SH-SY5Y cells, we studied the protein levels of mitochondrial dysfunction-related genes (PGC-1α, TFAM, COX4, and ERRα). In particular, the protein level of PGC-1α, a master regulator of mitochondrial biogenesis, is markedly elevated in 5 mM VPA-treated cells within 4 h (Figure 2B,C; *p* < 0.05). Protein levels of TFAM (at 1 mM and 5 mM) and COX4 (at 5 mM) change significantly with VPA treatment (Figure 2B,C; TFAM, *p* < 0.05, at 1 mM, *p* < 0.01 at 5 mM; COX4, *p* < 0.01 at 5 mM). However, ERRα levels decrease with 5 mM VPA (Figure 2B,C, *p* < 0.05).

### 3.3. Acute VPA Treatment Induced FOXO3a Expression in SY-SY5Y Cells

**Figure 3:**
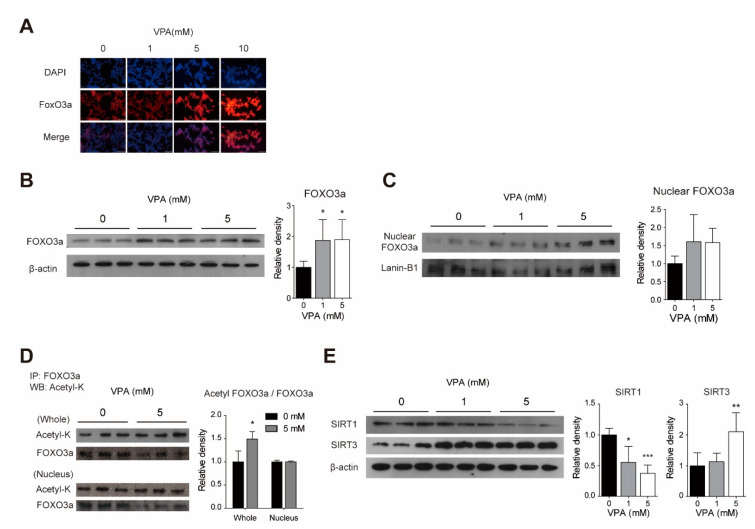
Acute VPA treatment induced FOXO3a expression in SY-SY5Y cells. VPA modulates the FOXO3a acetylation with the change of SIRT1 and SIRT3 protein levels in SH-SY5Y cells. Expression and location of the target protein were observed in cells treated with different concentrations (0–10 mM) of VPA for 24 h. (**A**) The location of Foxo3a was observed through immunocytochemistry. 4′, 6-diamidino-2-phenylindole (DAPI) was used to confirm nuclear location. Foxo3a was identified as red, and DAPI as blue fluorescence. Scale bar = 50 μm. (**B**) Expression of Foxo3a in the total protein extracted with RIPA buffer was confirmed by Western blot. β-actin was used as a loading control. (**C**) Nuclear proteins were extracted from the cells using the NEPER kit, and Foxo3a expression was confirmed by Western blot. Lamin-B1 was used as a loading control. (**D**) The proteins of whole lysate and nucleic fraction were obtained using the IP/Lysis buffer in PierceTMDirect IP Kit and NEPER Kit in SH-SY5Y cells. With these proteins, immunoprecipitation was performed using PierceTMDirect IP Kit and Foxo3a antibody. Expression of acetyl-K was confirmed by Western blot. Foxo3a was used as a loading control. (**E**) Expression of SIRT1 and SIRT3 in the total protein extracted with RIPA buffer was confirmed by Western blot. β-actin was used as a loading control. The level of protein expression was calculated by analyzing the band with the ImageJ program and normalized against the loading control expression level. Values represent the mean ± SD. (**B**,**C**,**E**) is *n* = 6 and (**D**) is *n* = 3. * *p* < 0.05, ** *p* < 0.01, and *** *p* < 0.001 with the 0 mM group.

We examined whether the protein level of FOXO3a, which is known to play an important role in autophagy associated with mitochondria, changes after VPA treatment using Western blot and immunocytochemistry. The immunocytochemistry results show that VPA treatment increases FOXO3a immunoreactivity in SH-SY5Y cells (Figure 3A). Consistent with the immunocytochemistry results, Western blot results also show that the level of FOXO3a protein increases after VPA treatment (Figure 3B, *p* < 0.05, at 1 mM and 5 mM). VPA treatment increases the amount of nuclear FOXO3a, but it is not statistically significant (Figure 3C, *p* = 0.20 at 1 mM, *p* = 0.16 at 5 mM).

### 3.4. VPA Modulates the FOXO3a Acetylation with Changes to SIRT1 and SIRT3 Protein Levels in SH-SY5Y Cells

We investigated whether the acetylation of FOXO3a, which is known to affect the subcellular localization and transcriptional activity of FOXO3a, is altered by VPA treatment through Western blotting. After 5 mM VPA treatment, when acetyl lysine levels are measured in the immunoprecipitation with anti-FOXO3 antibody, the protein level of total acetylated FOXO3a increases (*p* < 0.05), and the amount of acetylated FOXO3a in nuclear FOXO3a remains unchanged (Figure 3D). In addition, changes in the protein expression levels of SIRT1 and SIRT3 were confirmed by Western blotting. After VPA treatment, the amount of SIRT1 decreases (*p* < 0.05 at 1 mM, *p* < 0.001 at 5 mM) and the amount of SIRT3 increases (*p* < 0.01 at 5 mM) (Figure 3E).

### 3.5. VPA Treatment Induced Autophagy in SH-SY5Y Cells

**Figure 4:**
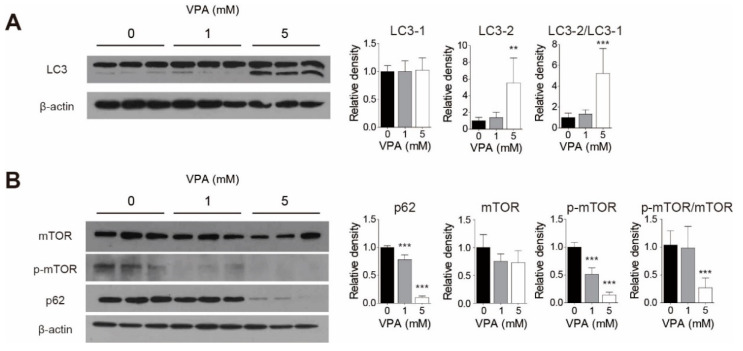
VPA treatment induced autophagy in SH-SY5Y cells. Total protein was isolated from the cells treated with different concentrations (0, 1, and 5 mM) of VPA for 24 h using RIPA buffer. (**A**) LC3, (**B**) mTOR, *p*-mTOR, and p62 expression were observed by Western blot. β-actin was used as a loading control. The level of protein expression was calculated by analyzing the band with the ImageJ program and normalized by the loading control expression level. Values represent the mean ± SD (*n* = 6). ** *p* < 0.01, and *** *p* < 0.001 with the 0 mM group.

To study whether VPA treatment induces autophagy in the SH-SY5Y cell line, we confirmed the protein levels of LC-3, a standard marker for autophagosomes, and p62, an autophagy substrate, by western blotting. We also measured the change in the phosphorylation of the mammalian target of rapamycin (mTOR), an important regulator of autophagy, using Western blotting. When 5 mM VPA was added, the LC3-II level and ratio of LC3-II to LC3-I increases (*p* < 0.01 and *p* < 0.001, respectively; Figure 4A). The protein level of p62 decreases when treated at VPA concentrations higher than 1 mM (Figure 4B; *p* < 0.001 at 1 mM and 5 mM). VPA decreases the level of phosphorylated mTOR at concentrations higher than 1 mM (Figure 4B: *p* < 0.001 at 1 mM and 5 mM) and significantly reduced the ratio of p-mTOR/mTOR (Figure 4B: *p* < 0.001). Our results demonstrated that VPA treatment induced autophagy in SH-SY5Y cells.

**Figure 5:**
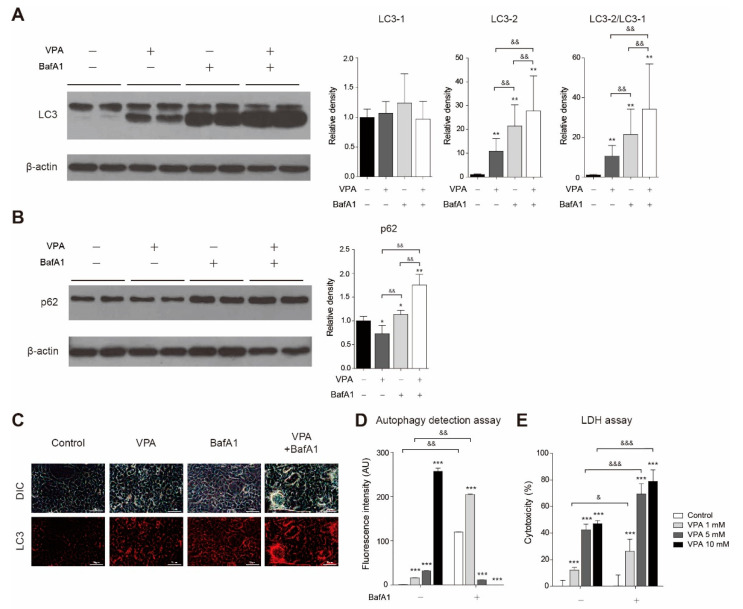
Bafilomycin A1 treatment blocked VPA-induced autophagic flux in SH-SY5Y cells. Total protein was isolated from the cells treated with/without 5mM VPA and/or 100nM bafilomycin A1 for 24 h using RIPA buffer. (**A**) LC3 and (**B**) p62 expression were observed by Western blot. β-actin was used as a loading control. The level of protein expression was calculated by analyzing the band with the ImageJ program and normalized by the loading control expression level (*n* = 6). (**C**) The location of LC3 was observed through immunocytochemistry. LC3 was identified as red. Scale bar = 50 μm. (**D**) Autophagy detection assay kit was used to observe changes in the amount of autophagic vacuole in cells treated with VPA and bafilomycin A1 for 24 h (*n* = 6). (**E**) LDH assay kit was used to observe the cytotoxicity in cells treated with VPA and bafilomycin A1 for 24 h (*n* = 6). Values represent the mean ± SD. ** *p* < 0.01, and *** *p* < 0.001 with the 0 mM group. & *p* < 0.05, && *p* < 0.01, and &&& *p* < 0.001 between groups.

We also tried to determine how disruption of autophagic flux caused by bafilomycin A1 affects LC3 and p62 levels changed by VPA. When we treated 5 mM VPA, the LC3-II level and the ratio of LC-3 II to LC3-I were significantly increased compared to the control group (Figure 5A; *p* < 0.01). When VPA and bafilomycin A1 were treated together, the LC3-II level and the ratio of LC-3 II to LC3-I were significantly increased compared to when VPA or bafilomycin A1 was treated alone (Figure 5A; *p* < 0.01 between VPA only group vs. VPA plus bafilomycin A1 group, *p* < 0.01 between bafilomycin A1 only group vs. VPA plus bafilomycin A1 group). The p62 level was significantly decreased compared to the control group after 5 mM VPA treatment (Figure 5B; *p* < 0.05). When VPA and bafilomycin A1 were treated together, the p62 level was significantly increased compared to when VPA or bafilomycin A1 was treated alone (Figure 5B; *p* < 0.01 between VPA only group vs. VPA plus bafilomycin A1 group, *p* < 0.01 between bafilomycin A1 only group vs. VPA plus bafilomycin A1 group). When VPA and bafilomycin A1 were treated together, it was also observed that the LC3 immunoreactivity was increased compared to when VPA or bafilomycin A1 was treated alone in the cells through immunocytochemistry (Figure 5C). Then, we measured the amount of autophagic vacuole in cells treated with VPA and bafilomycin A1 using an autophagic vacuole-specific dye. When we treated VPA, the green fluorescence autophagic vacuole detection signal was increased dose-responsively (Figure 5D; *p* < 0.001 at 1, 5, 10 mM). The fluorescent autophagic vacuole detection signal in the VPA 1 mM plus bafilomycin A1 group was significantly increased compared to the group treated with 1 mM VPA only (Figure 5D; *p* < 0.01) LDH assay results showed that the co-treatment of VPA higher than 5 mM and bafilomycin A1 induced high cytotoxicity (Figure 5E; 69.3 ± 7.6% at 5 mM VPA plus bafilomycin A1 group, 78.9 ± 8.7 % at 10 mM VPA plus bafilomycin A1 group). 

## 4. Discussion

In this study, we confirmed that VPA treatment affects FOXO3a protein expression and acetylation in the SH-SY5Y neuroblastoma cell line. In addition, it was confirmed that autophagy occurs with changes in FOXO3a expression.

FOXO3a is thought to be a promising candidate as a cancer biomarker or therapeutic target. Deregulation of FOXO3a activity has been reported to be associated with several malignancies [19,20,21]. In neuroblastoma, FOXO3a inactivation is essential for cell survival, and an increase in FOXO3a expression in SH-SY5Y cells potentiates apoptosis by PI3K/AKT inhibitors [10]. It has been suggested that a positive autofeedback loop contributes to elevated FOXO3a [22]. Furthermore, it has been reported that the transcription factor Sp1 is acetylated in response to oxidative stress by glutathione depletion in neurons. Treatment with histone deacetylase (HDAC) inhibitors, such as VPA, induces Sp1 DNA binding via acetylation [23]. Luciferase reporter assay revealed that a region corresponding to the SP1 binding sites located between −2000 bp and −1037 bp of the FOXO3a promoter was essential for transcriptional activity. Co-transfection of an SP1 expression vector with reporter constructs markedly increased luciferase activity in colorectal cancer cells [24]. Collectively, these results indicate that increased intra-nuclear acetylated SP1 drives FOXO3a gene transcription via oxidative stress or HDAC inhibition. FOXO3a levels can increase in mitochondrial toxin-treated oxidative stress-conditioned cells [25]. In addition, FOXO3a protects cells from oxidative stress by increasing the expression of manganese superoxide dismutase (MnSOD) [26].

Complex interactions exist in the regulation of FOXOs by various posttranslational modifications (PTMs). Various posttranslational modifications, including acetylation, regulate FOXO3a activity. When FOXO3a is deacetylated by SIRT3, nuclear localization and transcriptional activity of FOXO3a increases [27]. SIRT3-induced FOXO3a deacetylation has a great influence on mitochondria, including increasing mitochondrial biogenesis-related proteins (PGC-1α, TFAM) under oxidative stress [27]. This pathway is likely to affect the increase in mitochondrial biogenesis after VPA treatment. Among the proteins involved in mitochondrial biogenesis, ERRα levels were decreased in this study. In previous studies on the relationship between ROS signaling and ERRs, ROS induction lowered ERRα levels and increased ERRγ levels in various cell lines, which activated ROS defense mechanisms [28]. Further research is thus needed to determine whether changes in ERRα levels caused by VPA can affect antioxidant production.

Although it is known that SIRT1 also affects the function of FOXO3a by deacetylation, the expression level of SIRT1 protein in this study decreased with VPA treatment. Previous studies have reported that the levels of oxidative stress and SIRT1 have extensive crosstalk [29]. ROS can reduce the mRNA level of SIRT1 and increase SIRT1 proteasomal degradation [30,31,32]. The decrease in SIRT1 protein levels in this study may be due to increased oxidative stress caused by VPA. The increase in total acetylated FOXO3a may also be induced by decreased SIRT1, which is mainly distributed in the nucleus and cytoplasm [33].

In the present study, VPA induced autophagy with changes in FOXO3a expression in the neuroblastoma cell line. Autophagy is thought to play a role in helping tumorigenesis as a survival-promoting mechanism, but it also inhibits tumor proliferation in a context-dependent manner [34,35]. The dual PI3K/mTOR inhibitor PI-103-treated neuroblastoma cells have been reported to activate FOXO3a and trigger apoptosis [10]. Recent findings suggest that VPA enhances apoptosis by promoting autophagy with the decrease in AKT/mTOR phosphorylation in gliomas [36]. Phosphorylation by numerous protein kinases also regulates FOXO activity via a cytoplasmic-nuclear shuttle mechanism. AKT signaling, as the major regulator, is responsible for the phosphorylation of FOXO3a, which leads to the exclusion of FOXO3a from the nucleus, and subsequently inhibits the activity of FOXO3a [37]. Therefore, the increased nuclear FOXO3a levels and autophagy phenotypes in this study may be induced by decreased AKT phosphorylation of FOXO3 with VPA treatment.

In conclusion, this study showed that acute VPA exposure induces mitochondrial dysfunction and toxicity in neuroblastoma cell lines, increasing FOXO3a and acetylation. Further, VPA increased mitochondrial biogenesis and autophagy, which are mechanisms related to FOXO3a. The effects of VPA on neuroblastoma obtained in this study will broaden our understanding of the therapeutic mechanisms. Further studies on the mechanism of FOXO3a modulation and autophagy by VPA may therefore increase the possibility of using VPA and FOXO3a-related substances as neuroblastoma therapeutics.

## Data Availability

Data are contained within the article.

## Supplementary Materials

The following are available online at https://www.mdpi.com/article/10.3390/cells10102522/s1, Table S1: Primer sequences for target genes.
